# Challenging manifestations of ANCA-associated vasculitis treated with avacopan: two case reports

**DOI:** 10.3389/fimmu.2025.1717889

**Published:** 2025-12-08

**Authors:** Aglaia Chalkia, Anastasia Politi, Isavella Tryfonos, Christos Koutsianas, Harikleia Gakiopoulou, Dimitrios Vassilopoulos, Dimitrios Petras

**Affiliations:** 1 Nephrology Department, Hippokration General Hospital, Athens, Greece; 2 2nd Department of Medicine and Laboratory, Clinical Immunology - Rheumatology Unit, National and Kapodistrian University of Athens School of Medicine, Hippokration General Hospital, Athens, Greece; 3 1st Department of Pathology, National and Kapodistrian University of Athens, School of Medicine, Athens, Greece

**Keywords:** avacopan, ANCA-associated vasculitis, interstitial lung disease, heart, epididymo-orchitis

## Abstract

Anti-neutrophil cytoplasmic antibody (ANCA)-associated vasculitis (AAV) is a small-vessel vasculitis that frequently affects the kidneys and lungs. Avacopan, a C5a receptor inhibitor, has demonstrated efficacy as a glucocorticoid-sparing therapy, but data on rare or severe manifestations, such as interstitial lung disease (ILD), cardiac involvement, and epididymo-orchitis, remain limited. We report two patients with newly diagnosed AAV and severe kidney involvement requiring hemodialysis. Case 1, a 65-year-old man with MPO-AAV, presented with ILD with a Usual Interstitial Pneumonia (UIP) radiological pattern, cardiac dysfunction, ENT involvement, and peripheral neuropathy. Case 2, a 68-year-old man with PR3-AAV, presented with kidney disease and epididymo-orchitis. Both patients received corticosteroids, rituximab, cyclophosphamide, plasma exchange, and avacopan. Dialysis independence was achieved within the first month in both cases. In Case 1, left ventricular function normalized, and ILD showed radiological improvement over 6 months. In Case 2, epididymo-orchitis resolved completely within 1 month. Both patients achieved clinical remission with minimal glucocorticoid exposure. These cases provide insights supporting the safety and efficacy of avacopan in severe, multisystemic AAV, including rare manifestations, and highlight its potential to promote organ recovery and reduce glucocorticoid-related toxicity in high-risk presentations.

## Introduction

Anti-neutrophil cytoplasmic antibody (ANCA)-associated vasculitides (AAV) are characterized by necrotizing inflammation of small vessels and are classified into three clinical phenotypes: microscopic polyangiitis (MPA), granulomatosis with polyangiitis (GPA), and eosinophilic granulomatosis with polyangiitis (EGPA). AAV often present with multi-organ involvement, with kidney and lung manifestations being particularly common, occurring in 90–100% of patients with MPA and 50–80% of those with GPA (1).

Recent advances in the management of AAV have established rituximab or cyclophosphamide, in combination with a reduced-dose glucocorticoid regimen, as induction treatment, while avacopan, a C5a receptor inhibitor, has emerged as an effective glucocorticoid-sparing alternative (2, 3). The ADVOCATE trial demonstrated a higher rate of sustained remission at week 52 in the avacopan group compared with glucocorticoid therapy (4). Subgroup analyses revealed that this superiority persisted among patients with kidney involvement as well as those with lung or ENT manifestations (5, 6). However, evidence on the efficacy and safety of avacopan in rarer manifestations, such as heart involvement, epididymo-orchitis, or in more challenging clinical scenarios, such as interstitial lung disease (ILD), remains limited.

Here, we report two cases of AAV with severe kidney disease requiring hemodialysis at presentation: one with concomitant cardiac involvement and ILD, and the other with epididymo-orchitis. Both patients received avacopan as part of their induction regimen.

## Case 1 presentation

A 65-year-old man with a substantial history of heavy tobacco use and no known comorbidities was transferred from a regional hospital due to persistent fever, rapidly progressive kidney dysfunction, and dyspnea. He described a 2-month history of generalized weakness, fatigue, unintentional weight loss, and progressive right-sided hearing loss and hypoesthesia affecting both legs. On admission, laboratory results revealed acute kidney injury, with a serum creatinine of 5.8 mg/dL (compared with 1.7 mg/dL measured 10 days earlier), and a C-reactive protein (CRP) level of 121 mg/L (normal range <5 mg/L). Urinalysis demonstrated marked hematuria (>200 per high-power field) and dysmorphic red blood cells, while 24 h urine collection showed proteinuria of 388 mg/24 h. Extensive infectious workup—including serologies for *Brucella*, Legionella, *Coxiella*, *Morganella*, HIV, HBV, HCV, and interferon-gamma release assay—was negative, and both blood and urine cultures remained sterile. Immunological tests revealed IgG 2,663 mg/dL, IgG4–605 mg/dL, IgA 538 mg/dL, and rheumatoid factor 90.5 IU/mL. Antinuclear antibody (ANA) testing demonstrated a positive cytoplasmic speckled pattern at a titer of 1:160. Perinuclear ANCA (p-ANCA) were positive, with anti-myeloperoxidase (anti-MPO) antibody titers of 95.1 IU/L.

**Figure 1:**
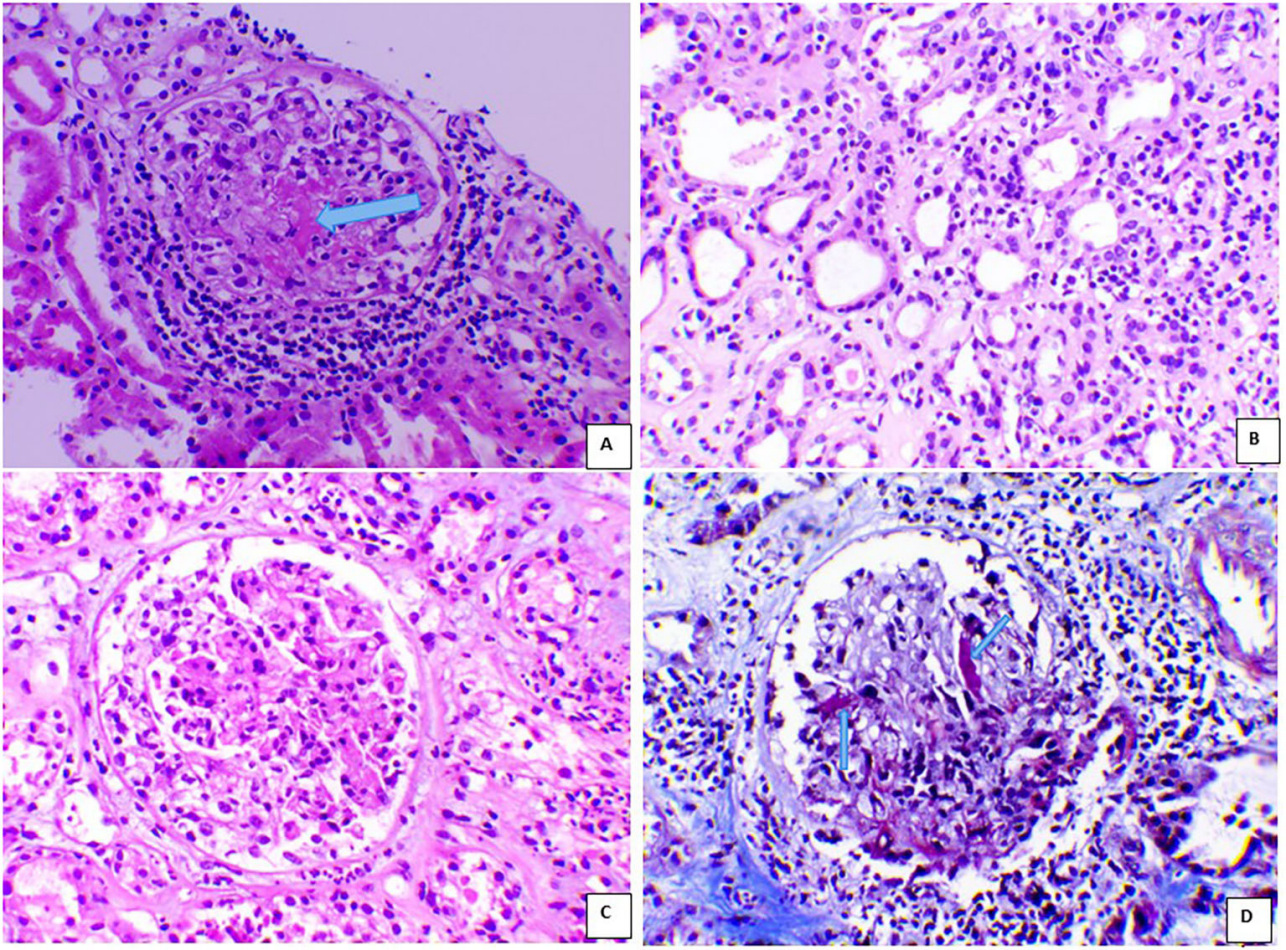
Case 1 and Case 2 kidney histopathology. **(A)** Segmental fibrinoid necrosis of the glomerulus (arrow) and periglomerular inflammation (HEx200). **(B)** Acute tubular injury and interstitial inflammation (HE x200). **(C)** Cellular crescent (HEx200). **(D)** Segmental fibrinoid necrosis of the glomerulus (arrow) (Masson x200).

**Figure 2:**
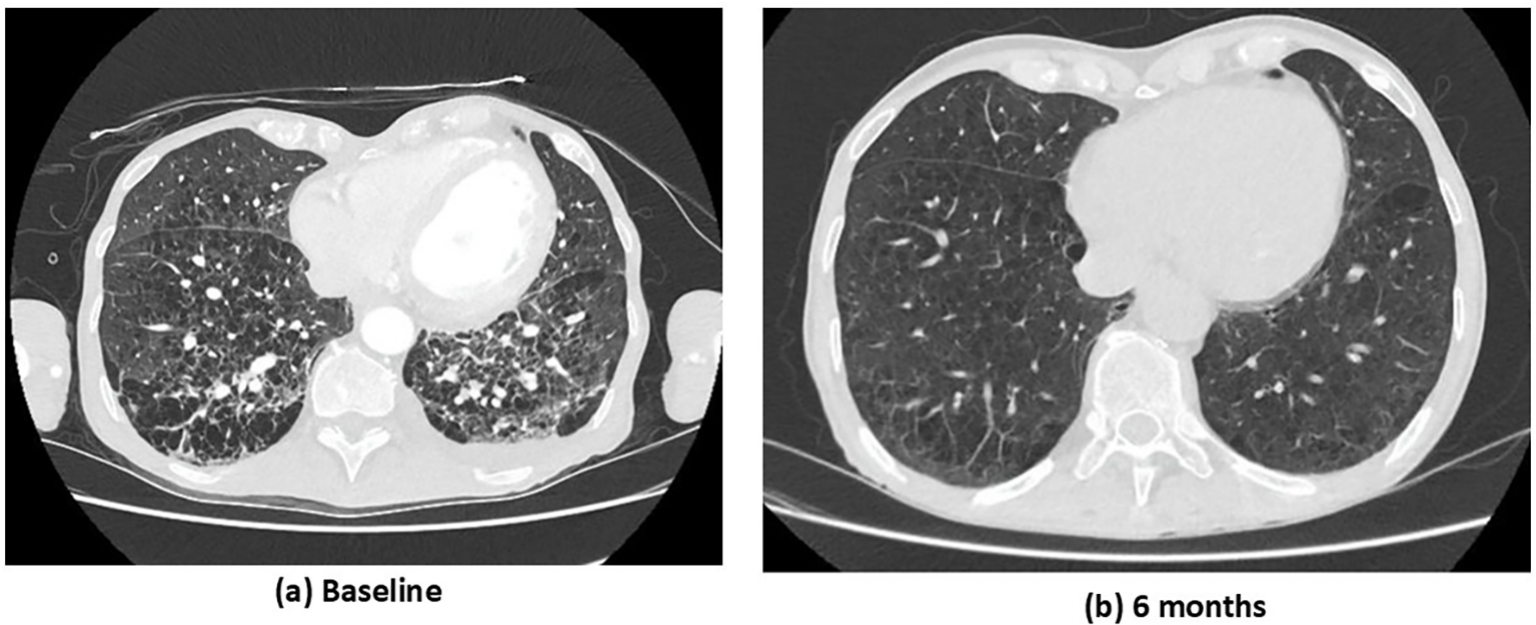
Case 1. CT chest study at baseline **(a)** and at 6 months following immunosuppressive treatment **(b)**. **(a)** Baseline: extensive bilateral ground-glass opacities and interlobular septal thickening, multiple areas of consolidation and reticular changes, and honeycombing in the lower lobes. **(b)** Six months: marked radiological improvement with near-complete resolution of ground-glass opacities and consolidations, significant reduction in interlobular septal thickening and reticular changes, and minimal residual reticulation with subtle parenchymal changes.

Kidney biopsy revealed pauci-immune necrotizing crescentic glomerulonephritis with severe acute tubular injury: 10% cellular crescents, 90% normal glomeruli, 10% interstitial fibrosis, and no global sclerosis (**Figures 1A, B**). Immunofluorescence evaluation showed mild IgM (1+/2+) positivity, while IgG, IgA, C3, C1q, C4, and κ and λ light chains were negative. The biopsy was classified as focal class by Berden’s classification and moderate risk according to the ANCA Kidney Risk Score (7). Systemic evaluation demonstrated multisystemic disease. High-resolution CT chest study revealed a usual interstitial pneumonia (UIP) pattern with extensive bilateral fibrotic thick-walled lesions and pleural-based honeycombing (**Figure 2A**). ENT involvement was indicated by thickening of the sphenoid sinuses and partial opacification of the right mastoid air cells on CT. On admission, serum troponin levels were markedly elevated (645 pg/mL) in the absence of chest pain. Transthoracic echocardiography revealed severely reduced left ventricular ejection fraction (LVEF 20%) with diffuse wall hypokinesia (compared with LVEF 65% ten days earlier). Coronary angiography excluded significant coronary artery disease. Cardiac magnetic resonance imaging (MRI) demonstrated myocarditis, with mid-septal interstitial fibrosis on T1 mapping and myocardial edema on T2 mapping. Clinical examination additionally revealed sensory deficits in both legs consistent with peripheral neuropathy.

The overall findings were consistent with MPO-AAV with severe multisystemic manifestations involving the kidney, lung, ENT region, heart, and peripheral nervous system (PNS). The Birmingham Vasculitis Activity Score (BVAS) was 41. Induction treatment included intravenous methylprednisolone (1 g × 3 days), followed by oral prednisolone (PEXIVAS reduced regimen), combined with rituximab (1 g × 2 doses 14 days apart) and cyclophosphamide (15 mg/kg every 14 days for 2 doses, RITUXVAS protocol). Given the severity of kidney impairment and hemodialysis dependence, the patient underwent seven sessions of plasma exchange. Considering his advanced age, severe kidney involvement, and high risk of steroid-related toxicity, avacopan (30 mg twice daily) was initiated 15 days after starting immunosuppression, alongside methylprednisolone tapering (total steroid duration 6 weeks). At 1 month, kidney function had partially improved (serum creatinine 5.3 mg/dL) without need for hemodialysis, and left ventricular systolic function had recovered significantly (LVEF 50%). At 3 months, further kidney improvement was observed (serum creatinine 3.9 mg/dL; estimated glomerular filtration rate [eGFR] 16 mL/min/1.73 m²), with normalization of systolic function (LVEF 55–60%). By 6 months, the patient achieved clinical remission. Rituximab was introduced for maintenance therapy, and avacopan was continued. A follow-up CT chest study revealed marked improvement in both the inflammatory and fibrotic pulmonary components (**Figure 2B**), and kidney function improved further (serum creatinine 2.9 mg/dL; eGFR 22 mL/min/1.73 m²; proteinuria 340 mg/24 h; no hematuria). Throughout the follow-up period, no adverse events such as neutropenia or hepatotoxicity were recorded. Only one serious infection occurred—a urinary tract infection—despite ongoing prophylactic antibiotic therapy for Pneumocystis jirovecii pneumonia (PJP).

## Case 2 presentation

A 68-year-old man presented to the emergency department with acute kidney injury (AKI) and progressive scrotal swelling over 10 days. His past medical history included hypertension, coronary artery disease, heart failure with reduced ejection fraction (HFrEF), atrial flutter previously treated with ablation, and psoriasis. In the preceding month, he had experienced generalized weakness, weight loss, and intermittent arthralgias. He had received a 10-day course of empiric oral ciprofloxacin for scrotal swelling without improvement.

**Figure 3:**
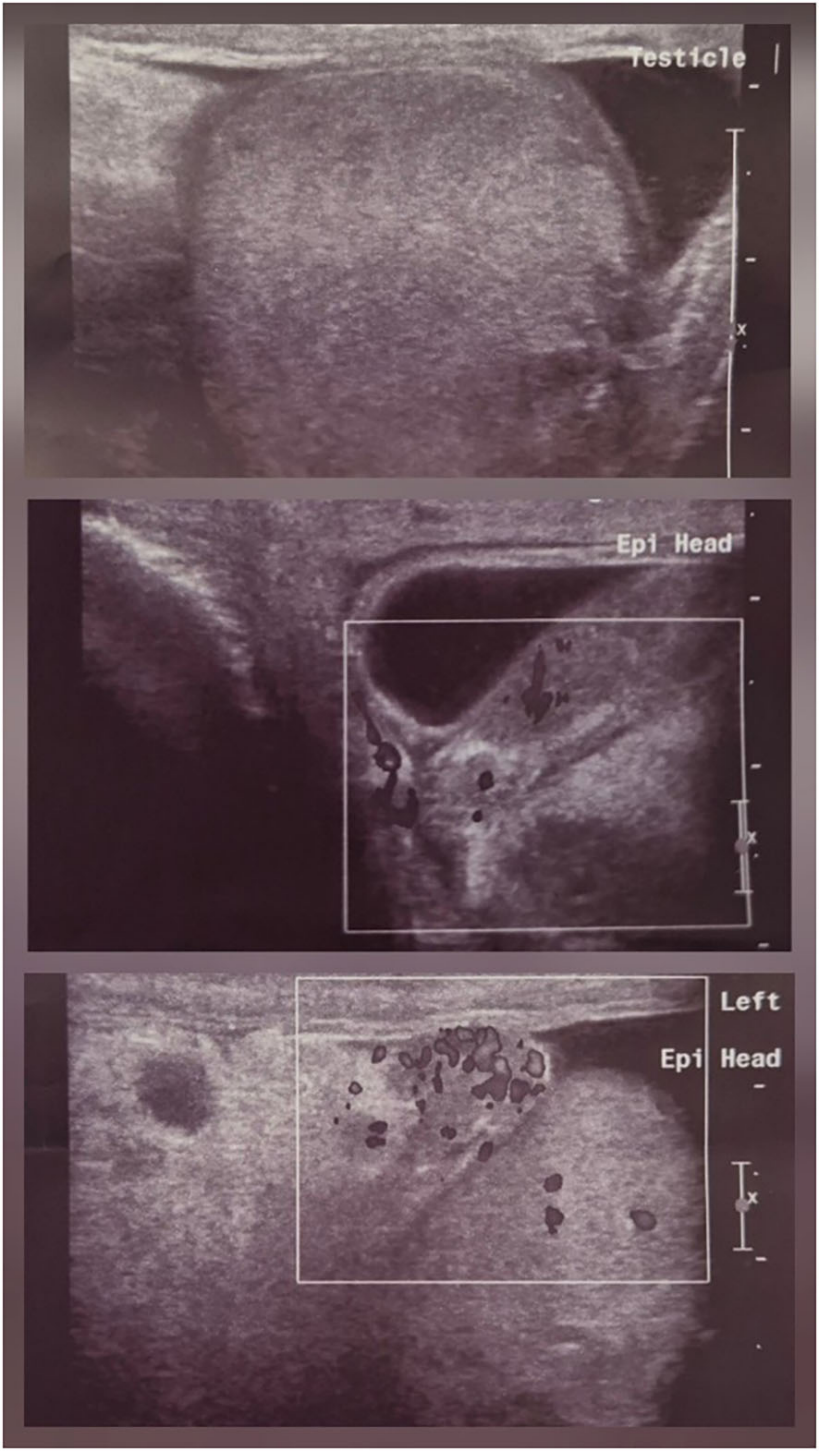
Case 2. Scrotal ultrasound. Enlarged, hypoechoic testis and epididymis with markedly increased vascularity.

On admission, his vital signs were stable: blood pressure 115/80 mmHg, oxygen saturation 95% on room air, heart rate 63 beats/min, and he was afebrile. Examination revealed fine basal crackles on lung auscultation, regular heart sounds without murmurs, absence of peripheral edema, and bilateral scrotal swelling and tenderness. There were no focal neurological deficits, palpable lymphadenopathy, ENT symptoms, or clinical signs of arthritis. Laboratory tests at admission revealed serum creatinine 5.3 mg/dL (baseline creatinine 1 mg/dL, and 4 days earlier 3.2 mg/dL), elevated C-reactive protein (CRP 160 mg/L; normal range <5 mg/L), and microscopic hematuria. Kidney ultrasonography showed normal-sized kidneys with increased cortical echogenicity. Scrotal ultrasonography revealed bilateral testicular enlargement with peritesticular fluid, preserved vascularity, and increased epididymal blood flow, consistent with acute epididymo-orchitis (**Figure 3**). Molecular testing of urethral secretions identified *Neisseria gonorrhoeae*; blood cultures were negative. Viral serologies (hepatitis B, hepatitis C, HIV) were negative. Complement (C3, C4) and immunoglobulin levels (IgA, IgG, IgM) were within normal limits. ANA testing was negative. Empiric treatment with ceftriaxone and levofloxacin was initiated, but no clinical or laboratory improvement was observed.

By day 7 of hospitalization, serum creatinine had peaked at 7.3 mg/dL, CRP remained high (140 mg/L), urinary sediment showed brown casts, and 24 h urinary protein excretion was 1.9 g/24 h. Bilateral scrotal swelling and tenderness persisted. The differential diagnosis included infection-related glomerulonephritis and ANCA-associated vasculitis (AAV) with kidney involvement and epididymo-orchitis, a rare manifestation of the disease. Further evaluation for systemic disease was undertaken. CT chest study demonstrated pulmonary emphysema and small bilateral pleural effusions. Transthoracic echocardiography revealed a left ventricular ejection fraction of 40%, with mild mitral regurgitation and no evidence of valvular vegetations. Immunologic testing revealed high-titer anti-proteinase-3 (PR3) antibodies (121.6 U/mL); ANA and anti-double-stranded DNA were negative. Kidney biopsy revealed 12.6% global sclerosis, 50% cellular crescents, 0% normal glomeruli, and 20% interstitial fibrosis, with pauci-immune immunofluorescence consistent with crescentic glomerulonephritis in keeping with AAV (**Figures 1C, D**). The biopsy was classified as crescentic type by Berden’s classification with a high ANCA Kidney Risk Score. Immunofluorescence evaluation showed mild C3 (1+/2+) and IgM (1+) positivity, while IgG, IgA, C1q, C4, and κ and λ light chains were negative.

A diagnosis of PR3-AAV with kidney involvement and epididymo-orchitis was established (BVAS 15). The patient received high-dose intravenous methylprednisolone pulses (1 g/day for 3 consecutive days, initiated on day 7 of hospitalization), followed by oral methylprednisolone per the PEXIVAS reduced-dose regimen. Rituximab (1 g × 2 doses 14 days apart) and cyclophosphamide (15 mg/kg every 14 days for 2 doses; RITUXVAS regimen) were administered. Due to rapidly progressive glomerulonephritis, seven consecutive sessions of plasma exchange were performed. Given his advanced age, severe kidney involvement, and high risk of steroid-related toxicity, avacopan (30 mg twice daily) was initiated 15 days after starting immunosuppression, alongside methylprednisolone tapering (total steroid duration 6 weeks). Intermittent hemodialysis was required but discontinued as kidney function improved. After 1 month, kidney function had significantly improved (creatinine 2.3 mg/dL), with further improvement at 3 months (creatinine 2.1 mg/dL; proteinuria 800 mg/24 h) and 6 months (creatinine 1.7 mg/dL; estimated glomerular filtration rate [eGFR] 41 mL/min/1.73 m²; proteinuria 800 mg/24 h; no hematuria). Notably, epididymo-orchitis began to improve within 2 days of steroid initiation and resolved completely within 1 month. During the total follow-up period, two episodes of leukopenia were recorded (nadir WBC 2,630/μL), both resolving after temporary discontinuation of avacopan. No serious infections occurred, and the patient remained on antibiotic prophylaxis for *Pneumocystis jirovecii* pneumonia (PJP).

## Discussion

We report two newly diagnosed cases of ANCA-associated vasculitis (AAV) with severe, difficult-to-treat manifestations. Both patients presented with advanced kidney involvement as well as additional severe manifestations. To date, the efficacy and safety of avacopan in AAV with cardiac involvement, interstitial lung disease (ILD), or epididymo-orchitis have not been described. Our report provides evidence supporting the use of avacopan in these clinical presentations.

Experimental models have demonstrated that activation of the complement alternative pathway is central to the pathogenesis of AAV. Systemic complement activation is evidenced by increased circulating levels of C5a, C3a, factor B, and the terminal complement complex (MAC), all of which decline with remission (8). Among these, C5a is a key mediator: by binding to its receptor (C5aR1), it strongly promotes neutrophil recruitment, activation, and degranulation. This pathogenic axis represents the therapeutic target of avacopan, an oral C5aR1 inhibitor. EULAR and KDIGO guidelines recommend that avacopan can be introduced as part of induction therapy in combination with rituximab or cyclophosphamide (2, 9). It is particularly indicated for patients with severe kidney involvement or those at high risk of glucocorticoid toxicity (3). The ADVOCATE trial showed that avacopan achieved higher rates of sustained remission at 12 months, improved quality of life, greater recovery of glomerular filtration rate (GFR), and fewer glucocorticoid-related adverse effects compared with a glucocorticoid regimen (4). In this trial, kidney disease was present in 81% of participants, all with a baseline estimated GFR (eGFR) >15 mL/min/1.73 m². In this subgroup, avacopan led to greater improvement in eGFR over 52 weeks (mean difference 3.2 mL/min/1.73 m²) compared with the glucocorticoid-treated group, and a more rapid reduction in urine albumin–creatinine ratio by the first month [absolute difference −40% (−53 to −22)] (6). The benefit was particularly notable in those with advanced kidney dysfunction at baseline, reaching an average gain of 5.5 mL/min in patients with eGFR <30 mL/min and up to 8.4 mL/min in those with eGFR <20 mL/min (10). The largest least-squares (LS) mean difference between the avacopan and prednisone taper groups was observed in patients with baseline eGFR 45–59 mL/min/1.73 m² (6.8 mL/min), followed by those with eGFR 15–29 mL/min/1.73 m² (5.6 mL/min) (6). Over the 52-week study period, 43.7% of patients in the avacopan arm improved by at least one eGFR category compared with 34.7% in the prednisone group. Real-world evidence further supports avacopan use in patients with kidney involvement and eGFR <15 mL/min/1.73 m², demonstrating preserved efficacy and no increase in adverse events, even in critically ill patients, such as those with hypoxic pulmonary hemorrhage (11, 12). In our report, both patients presented with end-stage kidney disease requiring hemodialysis; notably, each regained dialysis independence within the first month of treatment and demonstrated improvement of at least one eGFR category.

Pulmonary involvement in AAV covers a broad spectrum, ranging from diffuse alveolar hemorrhage and granulomatous lesions to nodules, cavitary forms, and ILD (13). ILD is increasingly recognized in a subgroup of patients with AAV, especially those with MPO-ANCA, and may even occur in the absence of vasculitis (14). Reported prevalence varies widely across studies, reflecting differences in methodology, patient populations, and disease severity. Unlike vasculitic pulmonary disease, which is characterized by capillaritis, ILD in AAV is driven by predominantly fibrotic processes affecting the interstitium, often with the development of scar tissue and the presence of lymphoid aggregates (15). Importantly, ILD in AAV is associated with substantially higher mortality compared with AAV without ILD, though survival may still be more favorable than in idiopathic pulmonary fibrosis (IPF) (16, 17). Prognosis appears to depend on factors such as the extent of lung involvement, underlying ANCA subtype, and radiologic ILD pattern. Despite its clinical impact, evidence-based treatment approaches for ILD in AAV remain poorly defined. In the ADVOCATE trial, 71 participants had lung involvement and were treated with avacopan (18). Among them, 52.1% presented nodules or cavities, 43.7% infiltrates, and 15.5% wheeze. At week 26, remission was achieved in 73.2% of patients in the avacopan group versus 66.2% in the prednisone taper group. By week 52, sustained remission occurred in 67.6% versus 53.5%, respectively (difference 14.1; 95% CI −1.8 to 30.0), and no active lung disease was reported. Although the effect of the complement C5a receptor inhibitor avacopan on ILD has not been studied, given that C5aR blockade may influence fibrotic and tissue-repair pathways in the kidney, it is plausible that avacopan could also mitigate pulmonary fibrosis (19). Notably, in our first case, ILD with a usual interstitial pneumonia (UIP) pattern showed radiologic improvement after 6 months of immunosuppressive therapy, including avacopan.

Cardiac manifestations in microscopic polyangiitis (MPA) are less common than in other forms of ANCA-associated vasculitis (AAV), such as eosinophilic granulomatosis with polyangiitis (EGPA) and granulomatosis with polyangiitis (GPA). Manifestations include pericarditis, cardiomyopathy, coronary artery disease, and heart failure (20). Experience with avacopan in this subgroup is very limited; in the ADVOCATE trial, only 6 participants (3.6%) with cardiovascular disease received avacopan, and no subgroup analysis was provided. Our case provides novel insights into the clinical efficacy and safety of avacopan in MPA-associated cardiac disease: the patient experienced recovery of left ventricular function within the first month, which remained stable over 6 months of follow-up. Testicular involvement is another rare manifestation of AAV, most often presenting as granulomatous inflammation of the testes or epididymis, and occasionally mimicking infectious or malignant processes. Evidence to guide treatment is sparse. In our patient, epididymo-orchitis resolved completely within 1 month of initiating therapy, with no recurrence during follow-up.

**Figure 4:**
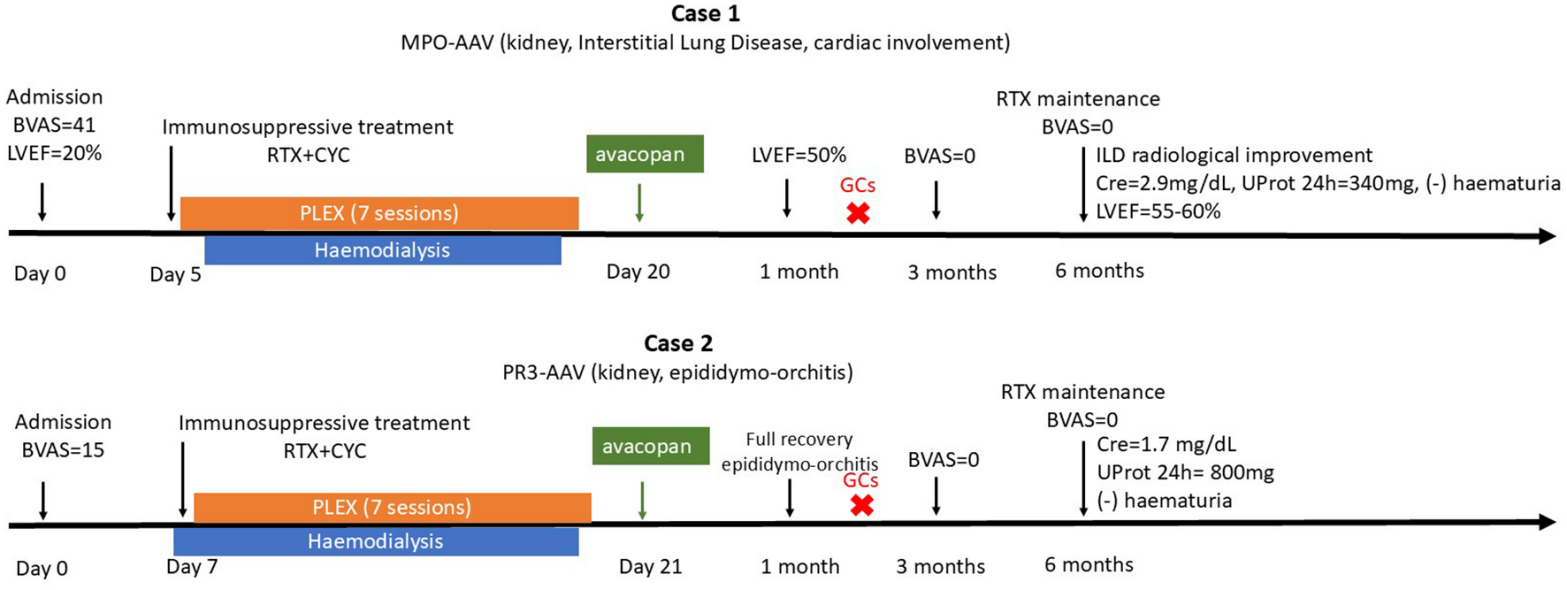
Timeline of Cases 1 and 2. AAV, ANCA-associated vasculitis, BVAS; Birmingham vasculitis activity score, LVEF; left ventricular ejection fraction, RTX; rituximab, CYC; cyclophosphamide, PLEX; plasma exchange, Cre; creatinine, GCs; glucocorticoids, UProt; urine proteinuria.

Despite both patients presenting with newly diagnosed, severe AAV and dialysis-dependent kidney failure, their clinical manifestations differed markedly. The first case, MPO-ANCA positive, exhibited predominant fibrotic and inflammatory features with ILD and cardiac involvement and was classified as Berden focal class, while the second, PR3-ANCA positive, presented with crescentic class and epididymo-orchitis as a rare manifestation. Both responded rapidly to combined immunosuppressive therapy, including avacopan, achieving dialysis independence and remission within 3 months. These findings highlight both the heterogeneity of AAV and the potential broad applicability of avacopan across distinct clinical phenotypes (**Figure 4**).

This report describes only two patients, and therefore the findings cannot be generalized. The favorable outcomes observed cannot establish a causal relationship between avacopan use and organ recovery, as both patients also received intensive immunosuppressive therapy, including corticosteroids, rituximab, cyclophosphamide, and plasma exchange. In addition, the relatively short follow-up period limits assessment of long-term efficacy, relapse rates, and potential delayed adverse effects. Larger prospective studies are needed to confirm the safety and efficacy of avacopan in severe and rare manifestations of AAV.

However, these cases highlight the potential of avacopan as a valuable therapeutic option in patients with AAV presenting with severe multiorgan involvement, including kidney, pulmonary (including ILD), cardiac, and testicular manifestations, providing clinical efficacy without increasing the risk of adverse events or serious infections. Both patients achieved significant kidney recovery with discontinuation of dialysis, alongside resolution of pulmonary and extrapulmonary disease, while avoiding long-term high-dose glucocorticoid exposure. Our observations add to the growing body of evidence supporting avacopan as an effective and safe alternative in complex and high-risk presentations of AAV.

## Data Availability

The original contributions presented in the study are included in the article/supplementary material. Further inquiries can be directed to the corresponding author.
